# Self-Patterning Tetrathiafulvalene Crystalline Films

**DOI:** 10.1021/acs.chemmater.3c01604

**Published:** 2023-10-11

**Authors:** St. John Whittaker, Merritt McDowell, Justin Bendesky, Zhihua An, Yongfan Yang, Hengyu Zhou, Yuze Zhang, Alexander G. Shtukenberg, Dilhan M. Kalyon, Bart Kahr, Stephanie S. Lee

**Affiliations:** † Department of Chemistry, Molecular Design Institute, New York University, New York, New York 10003, United States; ‡ Department of Chemical Engineering and Materials Science, Stevens Institute of Technology, Hoboken, New Jersey 07030, United States

## Abstract

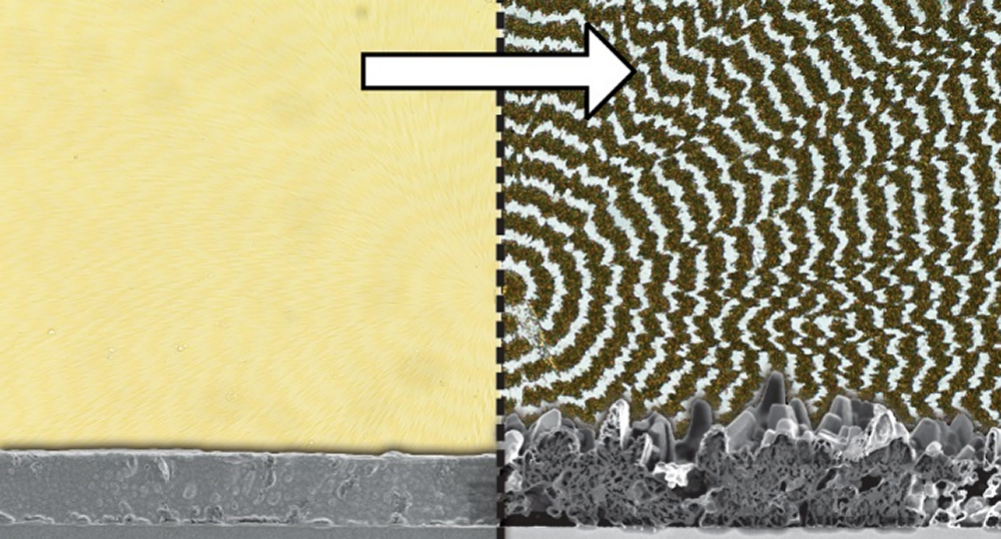

Tetrathiafulvalene (TTF) crystals grown from the melt are organized as spherulites in which helicoidal fibrils growing radially from the nucleation center twist in concert with one another. Alternating bright and dark concentric bands are apparent when films are viewed between crossed polarizers, indicating an alternating pattern of crystallographic faces exposed at the film surface. Band-dependent reorganization of the TTF crystals was observed during exposure to methanol vapor. Crystalline growth appears on bright bands at the expense of the dark bands. After a 24 h period of exposure to methanol vapor, the original spherulites were completely restructured, and the films comprise isolated, concentric circles of crystallites whose orientations are determined by the initial TTF crystal fibril orientation. While the surface of these outgrowths appears faceted and smooth, cross-sectional SEM images revealed a semiporous inner structure, suggesting solvent-vapor-induced recrystallization. Collectively, these results show that crystal twisting can be used to rhythmically redistribute material. Crystal twisting is a common and often controllable phenomenon independent of molecular or crystal structure and therefore offers a generalizable path to spontaneous pattern formation in a wide range of materials.

## Introduction

Arguably, the last century placed a premium on single crystals for structure determination and for integrated circuits requiring perfection, but often structure arises spontaneously when many crystals grow at the same time as a community. Spherulites^1^ are objects that can have crystallographically “impossible” optical symmetries such as that of the sphere group SO(3), the group of infinitesimal rotations about an origin; individual crystallites in some cases cannot be discerned in visible light, so infinitesimal rotations are effectively permissible. Even more spectacular are spherulites confined between glass slides that form disks but with concentric rings of optical contrast as a consequence of the twisting of the fibrils during growth about their radii. Helicoids are also crystallographically impossible forms, let alone spherical arrays of helicoids. Such so-called *banded spherulites* were shown to arise spontaneously in common molecular crystalline substances in the years before X-ray diffraction,^2^ only to be lost in the hegemony of X-ray crystallography with a systematic emphasis on single crystals. Banded spherulites reemerged in 1950 in synthetic polymers.^3−5^ Only recently have they been once again studied in crystals built from discrete molecules.^6−10^

As crystals change their orientations systematically, naturally, physical and chemical properties should alternate rhythmically along radii. Indeed, absorptivity,^11^ photoluminescence,^12^ electrical conductivity,^13^ linear dichroism,^14^ and linear and circular birefringence^15,16^ alternate between concentric bands of optical contrast. Here, we investigate whether the morphological evolution of a spherulitic film shows a dependence on the orientation of the underlying structure. In particular, we describe the reorganization and growth of patterned films of tetrathiafulvalene (TTF) when exposed to methanol vapor. Selective dissolution and recrystallization occurred on the dark and bright interference bands, respectively. After 24 h of methanol vapor exposure, TTF crystallites organized into isolated, polycrystalline ridges with spacings determined by the as-grown pitch. Crystal orientations in the original banded spherulite film determined those in the recrystallized ridges both along and perpendicular to the growth direction. Because pitches can vary from the submicrometer to millimeter length scale depending on the crystallization temperature, additive concentration, and other factors, ridge widths and spacings can be tuned accordingly.

## Experimental Methods

### TTF Film Fabrication

Tetrathiafulvalene (Sigma-Aldrich, >99% purity) and abietic acid (TCI, see discussion) were mixed in a 9:1 weight ratio using a mortar and pestle. ∼2 mg of the mixed powder was placed between two glass slides and melted at 120 °C. Slight pressure was applied to spread the liquid between the glass slides, and then the sample was immediately placed between aluminum blocks at room temperature. Complete crystallization occurred within a few seconds after sample transfer.

### Solvent Annealing

Melt-processed films were exposed to methanol solvent vapor by removing the top glass slide after crystallization and placing the sample above a methanol reservoir in a covered plastic Petri dish for times ranging from 0 to 64 h. For some experiments, methanol was drop-cast directly on TTF films to induce recrystallization within seconds.

### Film Characterization

Films were imaged using a combination of polarized optical microscopy (Olympus BX53 microscope), field-emission scanning electron microscopy (Carl Zeiss Merlin), and atomic force microscopy (Asylum Research Jupiter XR) in tapping mode using an AC55TS tip with a spring constant of ∼85 N/m. Orientation analysis of crystals in SEM images was performed using the Fiji plugin *OrientationJ* (EPFL, Switzerland).

### X-ray Diffraction

Transmission-mode X-ray diffraction patterns were collected at Brookhaven National Laboratory on Beamline 11-BM with an incident beam energy of 13.5 keV using a beam size of 100 μm^2^. A 2.6 × 2.6 mm grid was scanned in 26 100-μm steps in both the *x* and *y* directions, totaling 676 total diffraction patterns subsequently analyzed in Datasqueeze v3.0.20.

### Crystal Face Indexing

Angles between faces were measured by using the facet measurement function of Gwyddion AFM analysis software. By quantifying the vector normal to each facet of a single crystallite, the exterior angles between all facets were derived from the vector dot product. The angles between facets were tabulated and then quantitatively compared to all possible combinations of faces expected in the Mercury BFDH calculator (10 faces, 7 selected, 604,800 possible combinations). By calculating the percent difference between experimental AFM data and the theoretical exterior angle between planes from the single crystal structure, a difference-minimized set of faces was identified.

## Results and Discussion

**Figure 1:**
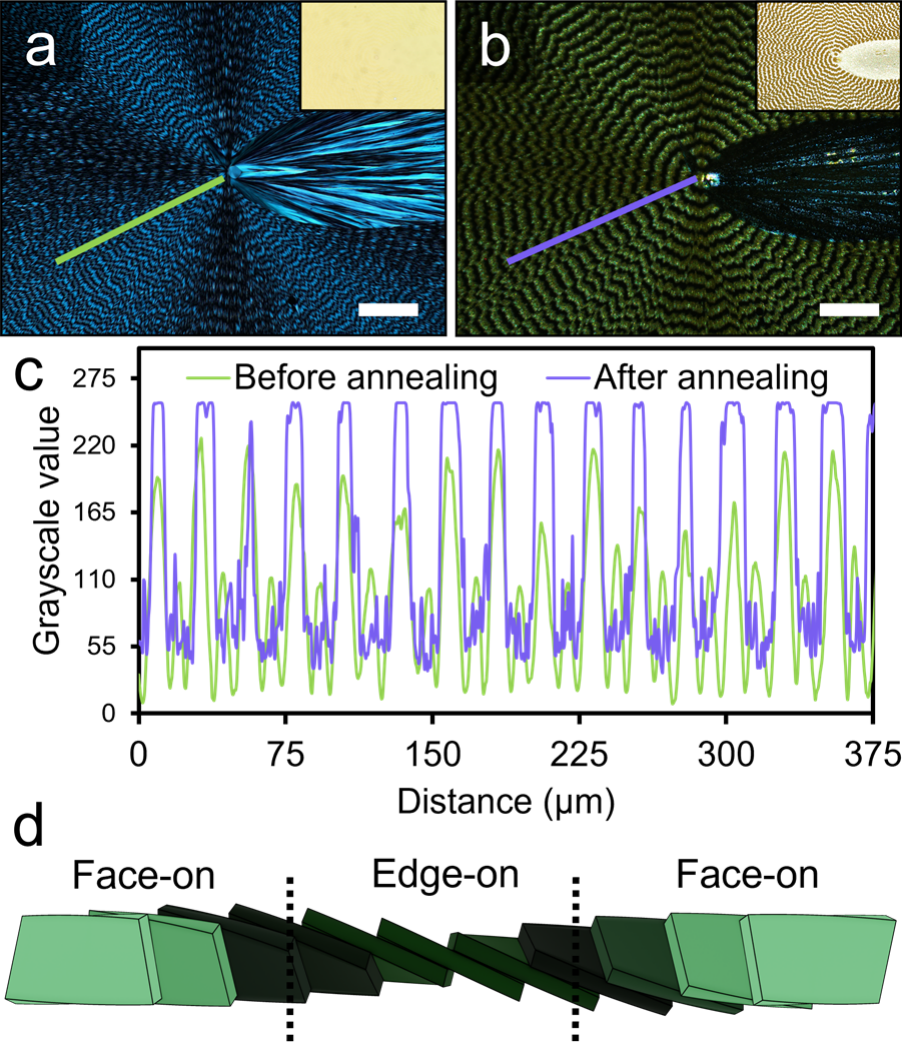
Transmission optical micrographs of the same banded TTF spherulite between crossed polarizers (a) before and (b) after 24 h of solvent vapor annealing. Inset micrographs were collected with unpolarized light. Scale bars: 100 μm. (c) Line profiles extracted from the grayscale micrographs along the colored lines in (a) and (b). (d) Illustration of face-on and edge-on orientations in a twisted fibril comprised of individual twisted plates. Individual radii are comprised of twisted crystallites and continually birth new fibrils through small angle branching. The complete form is a combination of lattice twisting and superposition of misoriented crystallites systematically.

Approximately 2 μm thick films of twisted TTF crystals were formed by melting TTF between two glass slides in the presence of 10 wt % abietic acid at 120 °C and then rapidly crystallizing the film at room temperature according to a previously published procedure.^17^ Abietic acid, which has been shown to promote crystal twisting in several molecular compounds, is thought to suppress spherulitic nucleation to achieve larger supercoolings.^18^ Larger supercoolings, in turn, promote the growth of finer fibrils with a greater propensity to twist. While abietic acid is a chiral molecule, it does not affect the TTF fibril twist sense—a circular retardance map of a TTF banded spherulite collected by Mueller matrix imaging revealed roughly equal populations of fibrils with left and right-handed twist senses (Figure S1).^15,17^ The diameter of 501 spherulites across 23 films was measured, which averaged 2.0 ± 0.6 mm. When viewed with unpolarized light, the optical micrograph (OM) of the TTF film appeared featureless, as displayed as an inset in Figure 1a. In a polarized optical micrograph (POM) collected with the sample between crossed polarizers, banded spherulites were observed, a telltale sign of crystal twisting (Figure 1a). These bands arise from continuously rotating crystal orientations as fibrils emanating from the spherulite center twist in concert with one another. Crystals exhibit orientation-dependent light absorption and refraction such that interference colors appear as periodic bands every ∼25 μm, i.e., the twisting pitch, *P*, and correspond to a 180° rotation of the crystal orientation about the growth direction. A Maltese extinction cross is observed in directions parallel and perpendicular to the polarizers, common for spherulites and other radially anisotropic bodies.^19−21^

The top glass slide was removed and the TTF film was solvent vapor annealed, a common method for improving the crystallinity of organic semiconductor thin films,^22−24^ in a closed petri dish with methanol. During this time, the film became opaque light yellow, indicating strong light scattering from the solvent vapor-annealed film. Figure 1b revealed the appearance of concentric bands spaced 25 μm apart, commensurate with twisting pitch measured before solvent vapor annealing, and indicative of a band-dependent structural change. Interference colors and the Maltese cross were still discernible in the corresponding POM, but with much weaker contrast. Differences in interference colors between Figure 1a,b are likely due to differences in film thickness. Other solvent vapors, including ethyl acetate, acetone, and tetrahydrofuran also induced spontaneous patterning (Figure S2), but pattern development was too rapid for detailed characterization. Methanol, which has a comparatively low vapor pressure and decreased solubility for TTF, was selected as the solvent vapor for further investigation.

Line profiles extracted from the grayscale images of each POM displayed in Figure 1c indicate that methanol vapor-induced structural relaxation of TTF banded spherulites into a more thermodynamically stable state occurs through recrystallization and dissolution of the bright and dark bands, respectively. X-ray diffraction patterns revealed that TTF crystals adopt the β phase upon melt phase crystallization and throughout the solvent vapor annealing process (CCDC refcode: BDTOLE02, *P*1̅, *Z*′ = 4, Figure S3)^25^ even though the β phase is metastable at room temperature.^26^ In contrast, complete dissolution and recrystallization of TTF in methanol result in crystals exclusively adopting the thermodynamically stable α phase. Band-specific recrystallization and dissolution are likely related to the morphological differences between crystals in each band. Individual helicoidal fibrils generally exhibit a platelike morphology with two wide faces corresponding to one crystallographic plane and four thin edges corresponding to the orthogonal crystal planes. As the plates rotate about the growth direction, they alternately present faces and edges at the film surface, typically referred to as “face-on” and “edge-on” orientations (Figure 1d).^27−29^ Face-on and edge-on crystal widths of 1.3 ± 0.5 and 0.38 ± 0.09 μm, respectively, were extracted from SEMs of TTF banded spherulite films (Figure S4). We speculate that because edge-on orientations have a higher density of step sites per unit area, they likely dissolve faster than face-on orientations.

**Figure 2:**
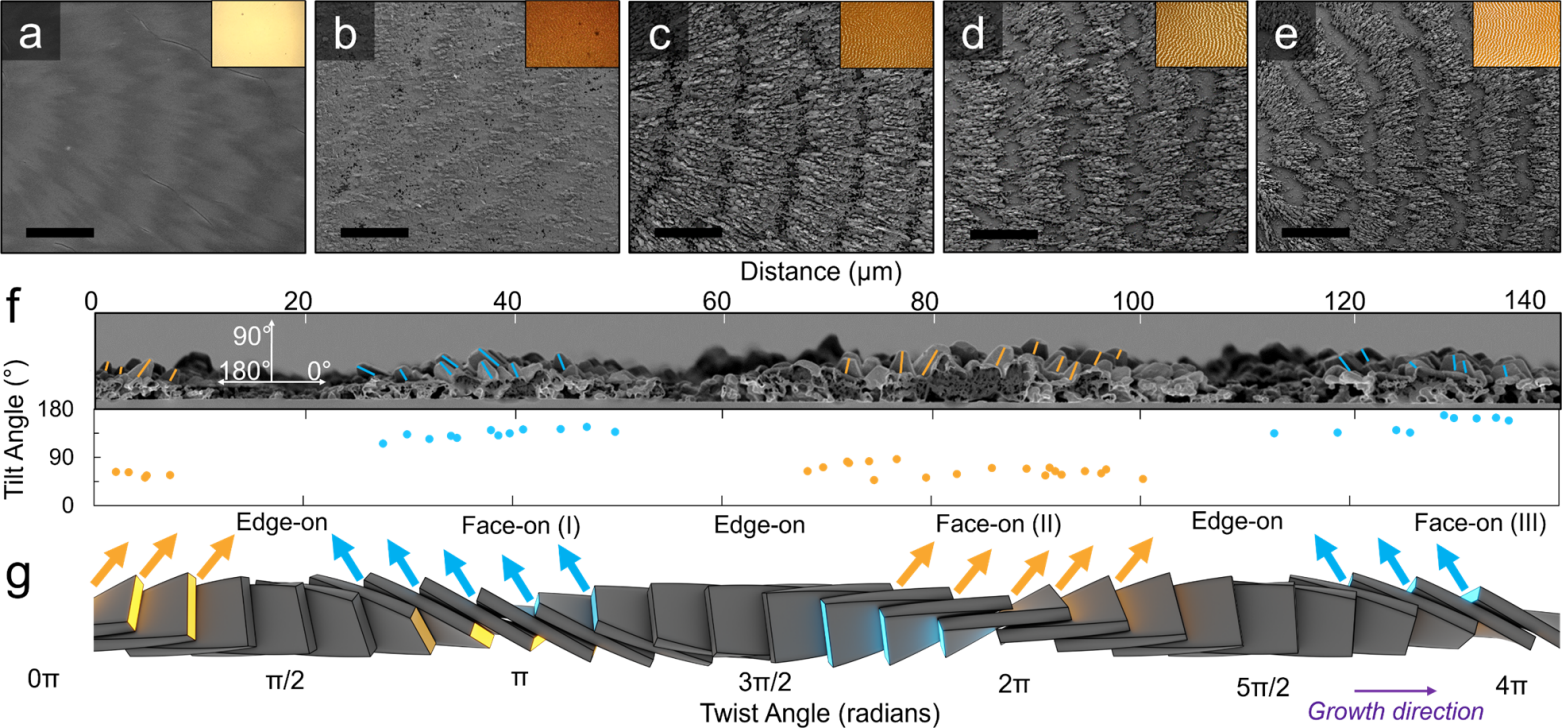
SEM images of TTF films solvent vapor-annealed for (a) 0, (b) 4, (c) 8, (d) 16, and (e) 24 h. Optical images inset. (f) SEM cross-section of a TTF film annealed for 24 h and corresponding plot of the crystallite tilt angle versus both distance and twist angle. Representative tilt angle measurements are overlaid in the SEM image as blue and orange lines. (g) A fibril of individual twisted crystallites (represented by twisted plates) to illustrate the relationship between twisting and tilt angles. Scale bars: 20 μm.

Time-dependent SEM images were collected on solvent vapor annealed films for times ranging from 0 to 24 h. As-processed banded TTF spherulites exhibited a smooth surface morphology with slight contrast between alternating bands (Figure 2a) corresponding to a ∼15 nm height difference between edge-on and face-on orientations as measured by AFM (Figure S5). Surface roughening was visible by 4 h of solvent vapor annealing, and the film darkened due to increased light scattering (Figure 2a–e insets). This roughening was periodic, with alternating bands forming ridges. The contrast between bands increased in both the SEMs and OMs with increasing solvent vapor annealing time until discrete ridges become separated by 24 h, after which no further restructuring was observed (Figures 2b–e and S6). Similar to the film in Figure 1, peak-to-peak ridge spacing matched the 25 μm twisting pitch in all of the annealed samples. After 24 h of annealing, individual crystallites within the ridges exhibited average lengths and widths of 4 ± 1 and 0.8 ± 0.3 μm across 180 measurements, respectively, with their long axes generally aligned parallel to the original spherulite growth direction. No evidence of abietic acid was found in either SEM images or X-ray diffraction patterns (Figure S7). It is possible that abietic acid resides in the interstitial spaces between TTF fibrils.

**Figure 3:**
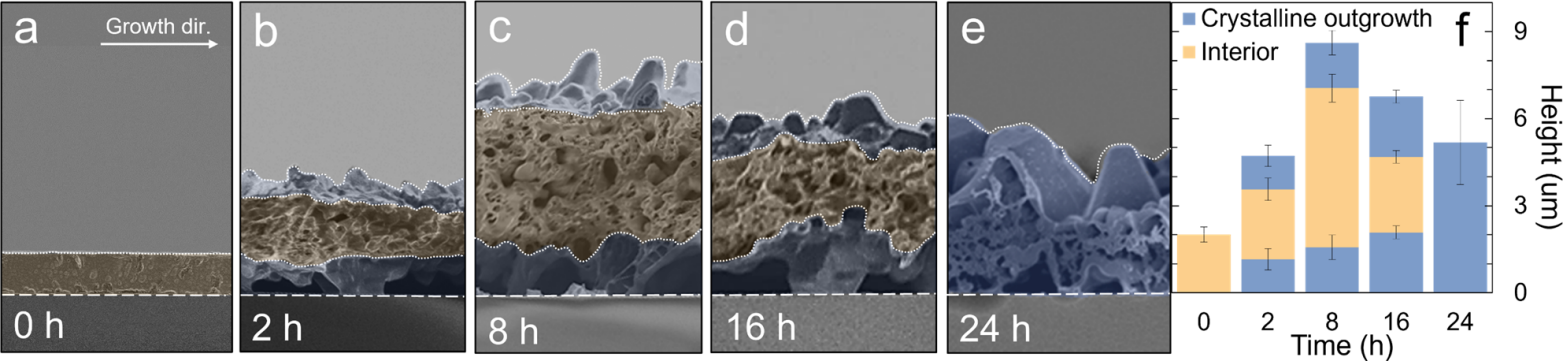
(a–e) SEM cross sections of TTF films comprising banded spherulites annealed for 0, 2, 8, 16, and 24 h, respectively. Films were qualitatively sectioned into crystalline outgrowths (blue) and interior (yellow) layers. Spherulitic growth direction is indicated by a white arrow. Scale bar is indicated in the *y*-axis of the bar graph in panel f. (f) Film thickness versus annealing time for crystalline outgrowths and interior layers. Error bars represent the standard deviation of at least 70 measurements collected over distances of at least 140 μm.

Figure 2f displays a cross-sectional SEM image of the 24 h annealed film shown in Figure 2e. The ridges and valleys are distinguishable, with ridge heights averaging 5 ± 1 μm (Figure 3e–f). A top-down SEM of the area shown in Figure 2f is included in the SI to help distinguish between ridges and valleys (Figure S8). Surprisingly, the micrometer-sized crystallites were porous despite their smooth polyhedral envelopes. These crystallites are tilted with the underlying banded substrate, as indicated by the blue and orange annotations on the SEM image. The tilt angles were quantified in the corresponding graph as a function of distance (Figures 2f and S9). Tilts alternated between 59° (colored orange) and 121° (colored blue) ± 15°.

A cross-sectional schematic of a twisted crystalline fiber is provided in Figure 2g to illustrate the relationship among ridge-valley spacing, fibril orientation, and crystallite tilt angle. Two ridges form with each π rotation of a twisted fibril in the regions labeled “Face-on (I)″ and “Face-on (II).″ While both regions adopt a face-on orientation from the top view, the exposed edges of the crystallites at the film surface are the trailing edge (highlighted blue) and leading edge (highlighted orange), respectively, as determined by the growth direction (purple arrow). Crystal growth likely occurs on these exposed edges, as evidenced by the alternating crystal tilts in Face-on (I and III) and Face-on (II) regions.

Figure 3a–e displays cross-sectional SEMs of films annealed for 0 to 24 h. The images were collected from the centers of the ridges. The as-crystallized film formed smooth, dense, ∼2 μm thick layers (Figures 3a and S10). After 2 h of solvent vapor annealing, the film height more than doubled to 4.7 ± 0.4 μm (Figure 3b). Three distinct layers were observed, a 2.4 ± 0.4 μm-thick film interior (shaded yellow) sandwiched between rough layers of crystalline outgrowths (blue). Crystalline outgrowths did not form in bands that eventually dissolved, and these bands were elevated from the underlying glass surface by the bottom crystalline outgrowths in adjacent bands (Figures S11 and S12). After 8 h of annealing, the ridge reached a maximum thickness of ∼7 μm, with a film interior of 5.3 ± 0.5 μm and crystalline outgrowths of 1.6 ± 0.4 μm. With annealing beyond 8 h, the total film thickness decreased due to a decrease in the interior. After 24 h of annealing, the film comprised only crystalline outgrowths, as summarized in Figure 3f. To rule out artifacts associated with TTF sublimation under vacuum during SEM imaging, we also collected atomic force microscopy (AFM) height maps of films at various annealing times. Film heights measured by AFM closely match those measured by cross-sectional SEM (Figures S13–S15), indicating that vacuum-induced sublimation during SEM was not responsible for the height changes. Instead, film thickness is a consequence of increased film porosity during solvent vapor annealing.

Crystallite growth from both the top and bottom film surfaces was apparent after 2 h of annealing (Figure 3b). Within ridges, the top and bottom crystallites were usually mirrored, i.e., left-leaning (relative to the substrate) top crystallites were countered by right-leaning bottom crystallites. This mirroring is most apparent after 8 h of annealing during which well-defined crystallites with distinct facets become visible. Crystallite tilt angles averaged ±59° (*n* = 67, Figures 3b–d, S9, and S11), consistent with a Face-on (II) region defined in Figure 2g. By 24 h of solvent vapor annealing, all the material from the original film assembled into isolated ridges comprising oriented crystallites.

These time-dependent cross-sectional SEMs suggest that methanol vapor both swells TTF films to create a porous interior and enables the diffusion of molecules from dissolving bands to recrystallizing bands. A strong orientational relationship between the original banded spherulite film and the recrystallized film is evident in both the appearance of a Maltese cross in recrystallized spherulites (Figure 1b) and the consistent crystallite tilts observed in alternating ridges (Figure 2f).

**Figure 4:**
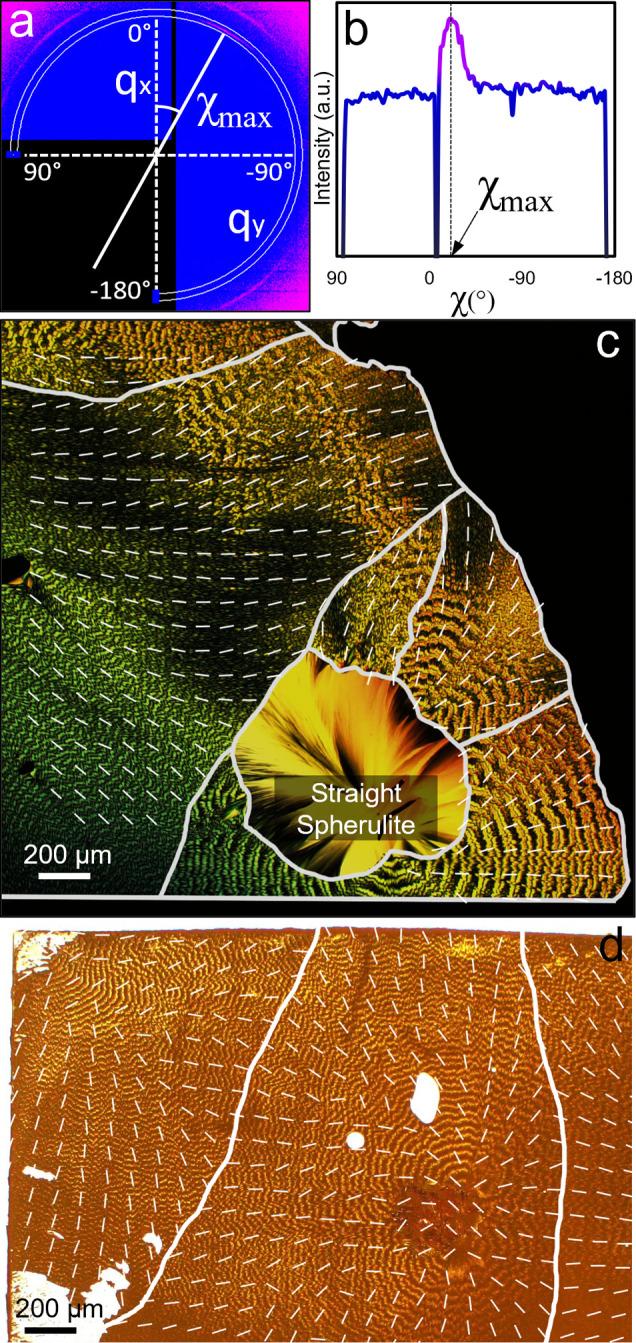
(a) Representative 2D X-ray diffraction pattern collected in transmission mode on a TTF film using a beam diameter of 100 μm. (b) Representative azimuthal line integration at *q* = 1.0 Å^–1^ (indicated by a white arc in a) versus χ, with χ_max_ labeled. (c) Lines with angles corresponding to χ_max_ overlaid on the POM of banded TTF spherulites. This reflection was not present in the diffraction patterns collected on the spherulite comprising straight crystals. (d) Lines with angles corresponding to χ_max_ overlaid on the OM of banded TTF spherulites annealed with liquid methanol for 4 s before rapid air drying.

Transmission mode X-ray diffraction analysis was conducted to further identify the crystal orientation in TTF-banded spherulite films before and after solvent vapor annealing. A scanning probe setup at the National Synchrotron Light Source II (Brookhaven, NY) Beamline 11-BM was used to collect a total of 676 diffraction patterns (Figure 4) in a 2.6 × 2.6 mm grid of 26 100 μm steps in each direction on an unannealed TTF film, which comprised mostly twisted crystals but with a spherulite of straight crystals in the bottom center. Azimuthal line scans along χ were extracted from each diffraction pattern at fixed *q* values, where peaks were observed. Figure 4a,b displays a representative azimuthal line scan collected at *q* = 1.0 Å^–1^_,_ which corresponds to the 020, 101̅, and 110 reflections from β-TTF crystals. Grazing incidence X-ray diffraction patterns indexed using Ocelot software were consistent with the twisting axis being the <010> direction (Figures S16 and S17). Spherulitic growth direction identification through this method is only possible due to slight variations in the growth direction relative to the substrate. Presumably, as twisted fibrils grow, the axis which they twist about is not perfectly straight, otherwise the {010} growth planes would be normal to the substrate, parallel to the incident beam, and unable to diffract. Bending can relax this condition.^6,17^

For each line scan, χ_max_, the χ value corresponding to the maximum intensity, was identified. Figure 4c displays a 2D map of χ_max_ values measured at *q* = 1.0 Å^–1^ represented by lines overlaid on top of an optical image corresponding to the scanned area of the TTF film. As observed in the figure, the orientation of these lines matches closely to the radial direction of the spherulites, indicating that this reflection corresponds to the spherulitic growth direction. A solvent-annealed sample was analyzed in the same way, and it was found that the overall diffraction pattern was maintained so that the reflection at *q* = 1.0 Å^–1^ closely aligned with the original spherulitic growth direction, despite the formation of crystalline outgrowths (Figure 4d). Transmission X-ray diffraction experiments confirm the close crystallographic relationship between the unannealed TTF twisted fibrils and crystallites formed during solvent vapor annealing. However, the beam size of 100 × 100 μm^2^ was too large to resolve changes in crystal orientations within individual bands.

**Figure 5:**
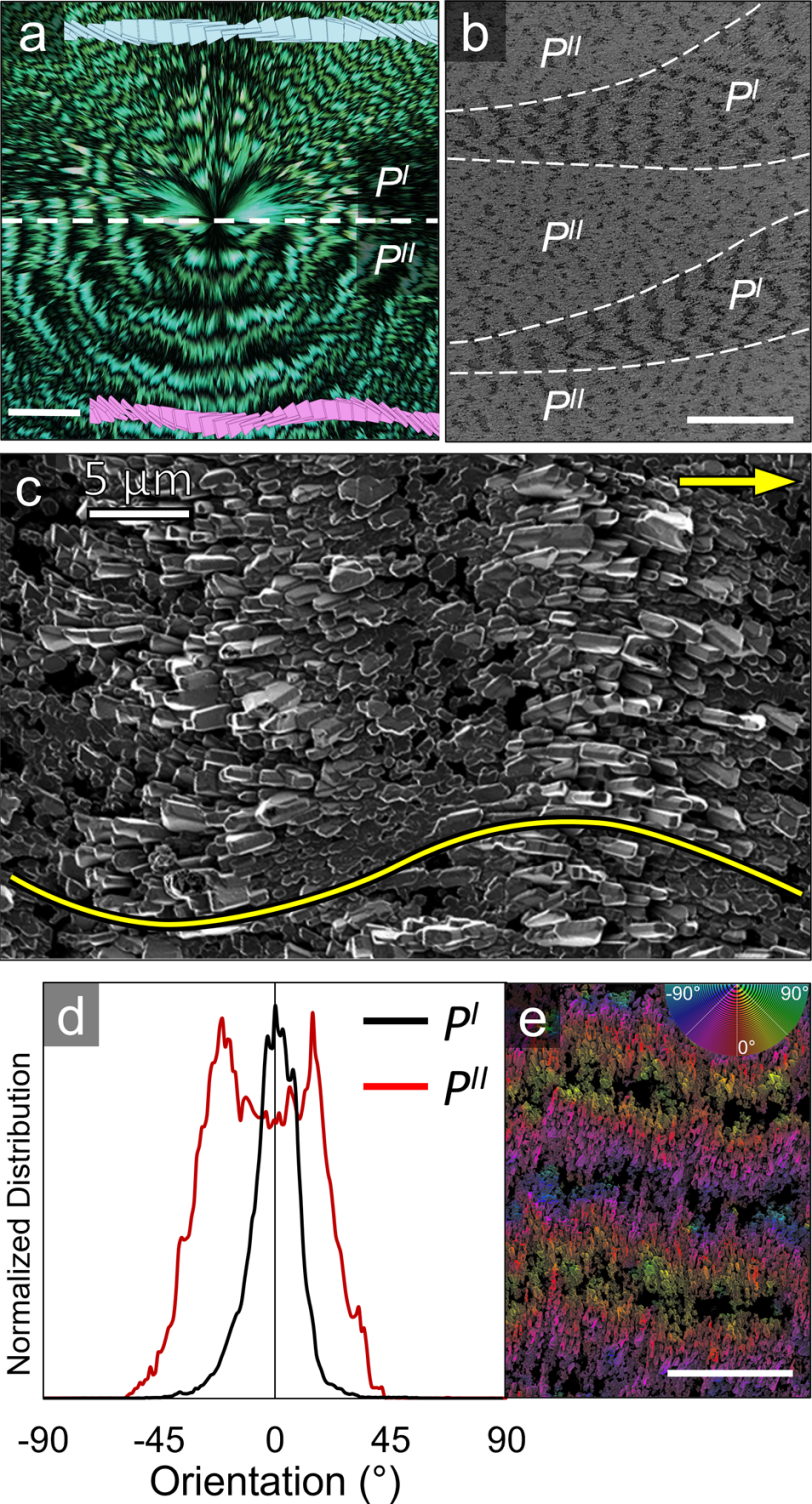
(a) POM of a banded TTF spherulite composed of *P*^*I*^ and *P*^*II*^ sections with a facsimile of corresponding twisted crystal fibers (not to scale). (b) SEM of film that was solvent vapor annealed for 8 h with both *P*^*I*^ and *P*^*II*^ labeled zones outlined with white dashed lines. (c) SEM of *P*^*II*^ spherulite solvent vapor annealed for 48 h. (d) Orientation analysis of *P*^*II*^ and *P*^*I*^ regions, analyzed with the Fiji plugin *OrientationJ* (EPFL, Switzerland). (e) SEM of *P*^*II*^ region analyzed in (d) and legend depicting hue assignment to orientation. Scale bars: 25 μm.

As discussed in a prior publication,^17^ two morphologies of banded TTF spherulites coexist, *P*^*I*^ and *P*^*II*^, in which the individual fibrils are respectively twisted, and bent and twisted. Here, only *P*^*I*^ spherulites have been described (Figure 1a). *P*^*II*^ spherulites lack a defined Maltese extinction cross and instead exhibit complex extinction when the spherulite radii are oriented between the crossed polarizers,^17^ which is a consequence of concomitant bending while twisting as illustrated in Figure 5a.^20^ We could not examine the bending morphlogy in unannealed films because of the limited contrast in the SEMs. However, as a consequence of the close crystallographic relationship between crystalline outgrowths formed during annealing and the underlying orientation of TTF crystals within the banded spherulites, solvent vapor annealing provides an opportunity to indirectly visualize the fibril morphology in *P*^*II*^ spherulites.

Figure 5b displays an SEM image of a film with both *P*^*I*^ and *P*^*II*^ sections that were solvent vapor-annealed for 8 h. Ridges were apparent in both the *P*^*I*^ and *P*^*II*^ regions, but the valleys between the ridges in the *P*^*II*^ regions were generally less defined than those in the *P*^*I*^ regions. This lack of distinct boundaries between ridges is likely reflective of the larger disorder at band edges in *P*^*II*^ compared to that in *P*^*I*^ regions (Figure 5a). Individual crystalline outgrowths from ridges have similar sizes and shapes between the *P*^*I*^ and *P*^*II*^ regions, but the progression of horizontal orientations on individual ridges separates *P*^*II*^ from *P*^*I*^. In *P*^*I*^ regions, crystalline outgrowths are generally oriented with their long axes parallel to the radial direction of the spherulite (Figure 2). In *P*^*II*^ regions, on the other hand, the long axes of the crystalline outgrowths splay about the radial direction along a sinusoidal pattern, as displayed in Figure 5c by the yellow arrow and sine wave, respectively. The angle between the crystallite long axes and the radial direction is largest at the ridge edges, while crystallites in the center of the ridges are more closely aligned with the radial direction. The pattern creates an S-shape in crystallite orientations spanning two ridges, so that the angles at the outer edges of the two ridges match, as well as the crystallites on the inner edge valley in the image center.

The serpentine pattern of the in-plane crystal orientations continues across the entire *P*^*II*^ spherulitic region. The Fuji plugin *OrientationJ* was used to quantify different crystallite orientations on a film that was annealed for 48 h (Figure 5d, e), showing crystallites oscillated ±17.5° on average relative to the spherulitic growth direction. To visualize this pattern, the color survey function of *OrientationJ* was used to assign hues to individual crystallites based on individual orientations (Figure 5e). The orientation pattern progresses from a maximum misalignment, with crystallites colored either green or blue at the edge of ridges, to crystallites colored red, which lie in the middle of the ridges, marking the apex of the turn back to ±17.5°. Comparatively, the *P*^*I*^ crystallites remain aligned along the spherulitic growth direction.

**Figure 6:**
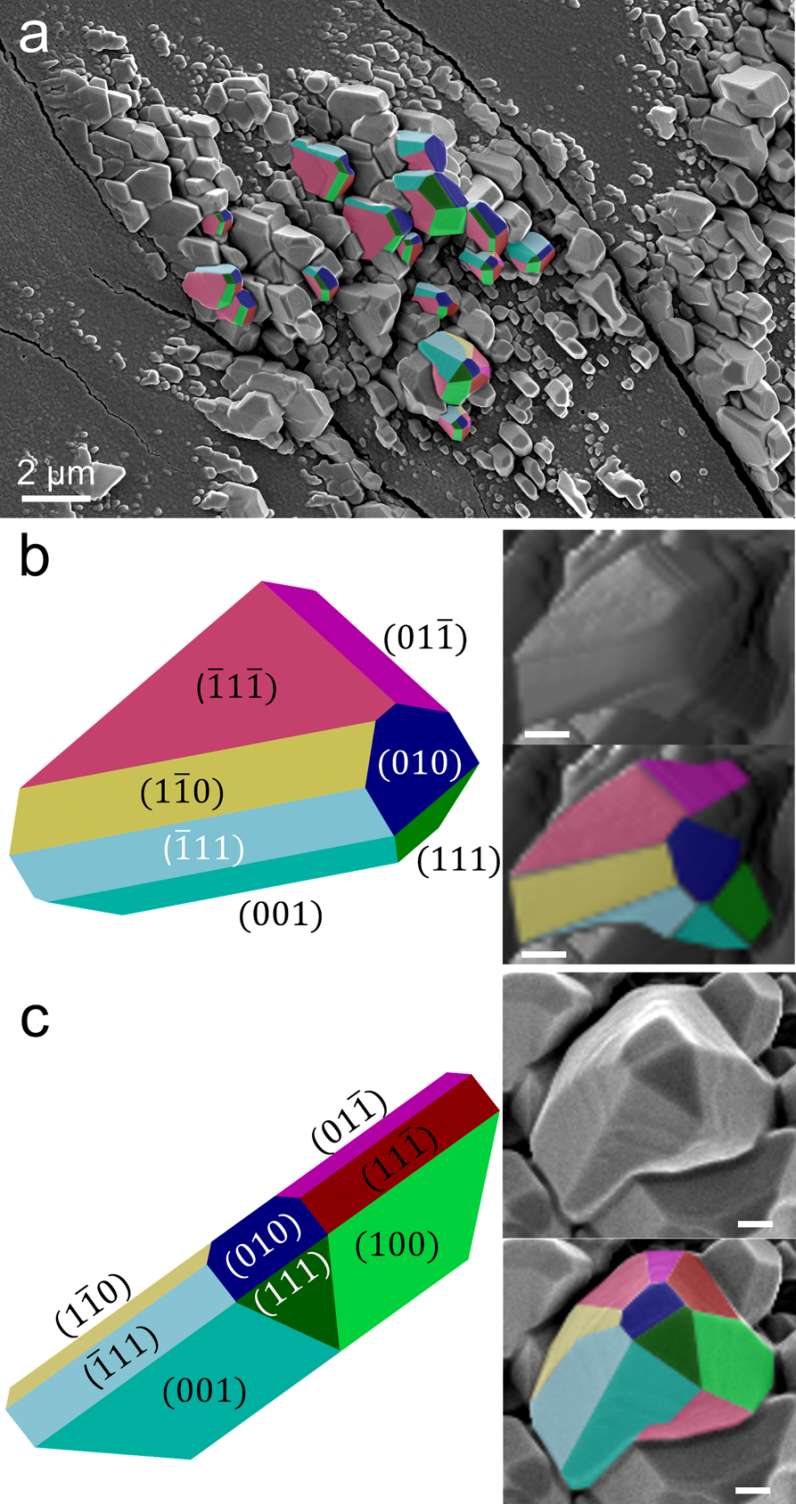
(a) SEM of crystallites liquid annealed for 4 s with false color matching idealized morphology facet color. (b) Simulated morphology of β-TTF crystallite and corresponding AFM amplitude retrace of a film solvent vapor annealed for 24 h. (c) Idealized morphology of crystallite in (a) and corresponding SEM. Scale bars: 500 nm.

In both the *P*^*I*^ and *P*^*II*^ regions, the crystalline outgrowths are faceted, reflective of single crystal domains. Upon close examination, similar geometric shapes can be identified on many of the crystalline outgrowths, as highlighted in Figure 6a. Single crystal morphologies reflect the unit cell symmetry, with angles between crystal faces corresponding to angles between (*hkl*) planes. Angles between individual crystallite faces were extracted from AFM height maps and matched to angles between (*hkl*) planes predicted by the single crystal structure of β-TTF (Figure S18).^25^ Out of more than 600,000 possible combinations of (*hkl*) planes examined, the set of planes whose angles with one another most closely match the experimentally measured angles between crystal faces were identified to be the (010), (1̅11̅), (011̅), (11̅0), (1̅11), (001), and (111) faces. Crystal morphologies were constructed in *JCrystal* based on this set of planes. By changing the relative areas of the crystal faces at fixed angles determined by the β-TTF unit cell, we identified different crystal shapes matching those observed in AFM and SEM images. Two such examples are provided in Figure 6b,c. For both crystallites, the (010) face is exposed at the top of the crystallite and adopts a trapezoid shape. Elongating the idealized crystal morphology in Figure 6b along the <010>, <111>, and <11̅1> directions and reducing the resulted in the crystal morphology displayed in Figure 6c. This morphology difference would result from slightly slower growth rates along the <010>, <111>, and <11̅1> directions in the former compared to the latter crystal.

By combining the crystal face indices with the observation of periodic crystal tilts in Figure 2 and the presence of mirrored crystallite growth between the top and bottom surfaces in Figures 3, S5, and S6, it appears that recrystallization proceeds from the exposed (010) faces of the original twisted fibrils, highlighted in orange and blue in Figure 2g. We can also assign the wide faces of twisted fibrils to the (11̅1) face (pink) and the narrower edges to the (1̅11) and (111̅) faces (light blue and red, respectively). Expression of the faces present in Figure 6b and c can account for nearly all of the crystallite morphologies observed in the SEM image in Figure 6a.

## Conclusions

The alternating bands in TTF twisted crystal spherulites are compositionally equivalent and distinguishable only when viewed between crossed polarizers. Spontaneous self-patterning of uniform films of banded TTF spherulites into ordered ridges and valleys follows methanol solvent vapor annealing. Band-dependent reorganization during solvent vapor exposure is a consequence of crystal-face-dependent surface energies. This mechanism of self-patterning is distinct from other materials, such as block copolymers, in which assembly directors (e.g., large enthalpies between polymer blocks) must be embedded into the chemical structure of the materials themselves. Looking forward, self-patterning organic electronic active layers present a low-cost strategy to form isolated semiconductor wires in order to reduce device crosstalk and leakage current that degrade device performance. Further control over wire placement could be achieved through the use of polymer molds to guide crystallization from the melt and collimate spherulitic bands.^30^ Because crystal twisting is expected to occur in at least one-third of all molecular compounds, the findings herein present a generalizable patterning method that does not require chemical synthesis.

## Supporting Information Available

Powder diffraction patterns, SEM images of TTF films, AFM height profiles, illustration of twisted and straight crystals, indexed 2D X-ray diffraction pattern, details on crystal face indexing (PDF)

## ABBREVIATIONS

TTF: tetrathiafulvalene
POM: polarized optical micrograph
SEM: scanning electron microscopy
AFM: atomic force microscopy
GIWAXS: grazing incidence wide-angle X-ray spectroscopy
