# Supplementary material for: Self-Patterning Tetrathiafulvalene Crystalline Films
Source: Chem Mater. 2023 Oct 11;35(20):8599–606. doi: 10.1021/acs.chemmater.3c01604 (PMC10601475; doi:10.1021/acs.chemmater.3c01604)
Supplement: Supplementary file 1 — cm3c01604_si_001.pdf [file cm3c01604_si_001.pdf]

SELF-PATTERNING TETRATHIAFULVALENE CRYSTALLINE FILMS

St. John Whittaker,[a] Merritt McDowell,[a] Justin Bendesky,[a] Zhihua An,[a] Yongfan Yang,[a] Hengyu Zhou,[a] Yuze Zhang,[b] Alexander G. Shtukenberg,[a] Dilhan M. Kalyon,[b] Bart Kahr,[a] Stephanie S. Lee*[,a]

[a]Molecular Design Institute, Department of Chemistry, New York University, New York, NY 10003, USA

[b]Department of Chemical Engineering and Materials Science, Stevens Institute of Technology, Hoboken, NJ 07030, USA

*stephlee@nyu.edu

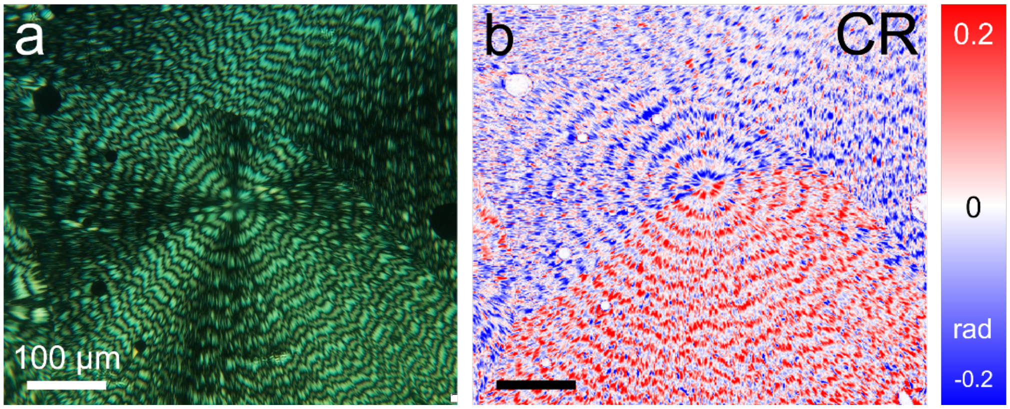

Figure S1: a) POM of TTF banded spherulite melt processed as a 9:1 mixture by weight with abietic acid. b) Circular retardance image of the same spherulite showing two zones of right and left handed heilicoidal crystal fibrils.

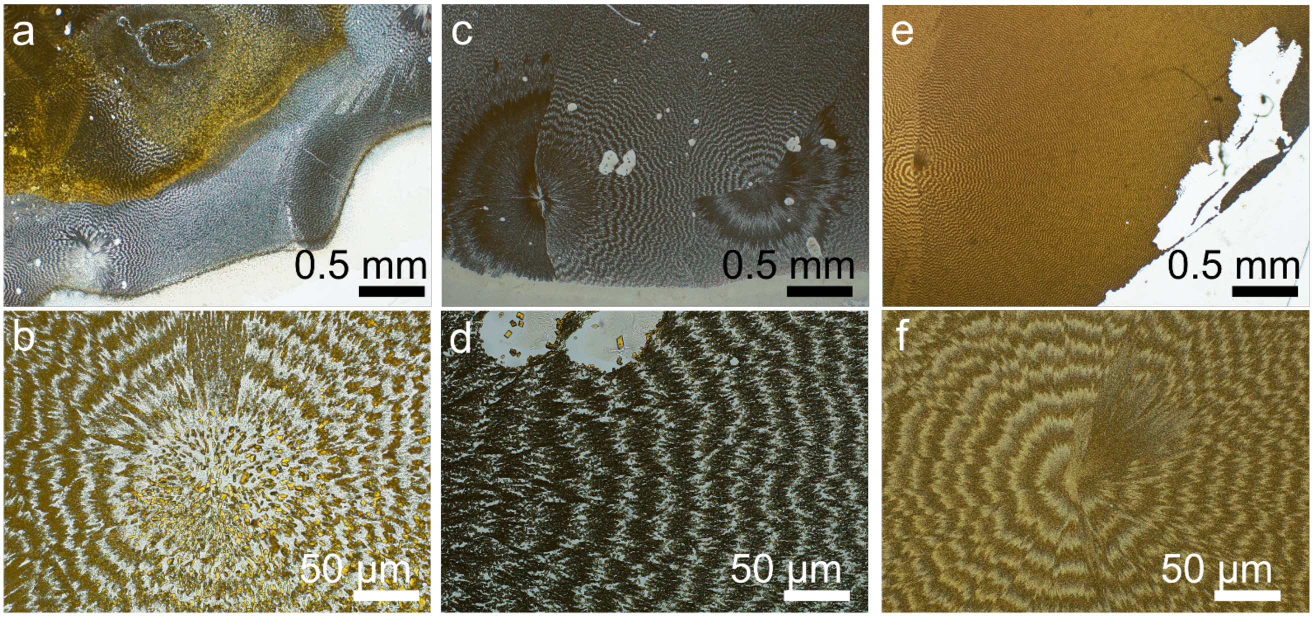

Figure S2: OMs of banded TTF films solvent vapor annealed with; a, b) ethyl acetate, c, d) acetone, and e, f) tetrahydrofuran at two magnifications.

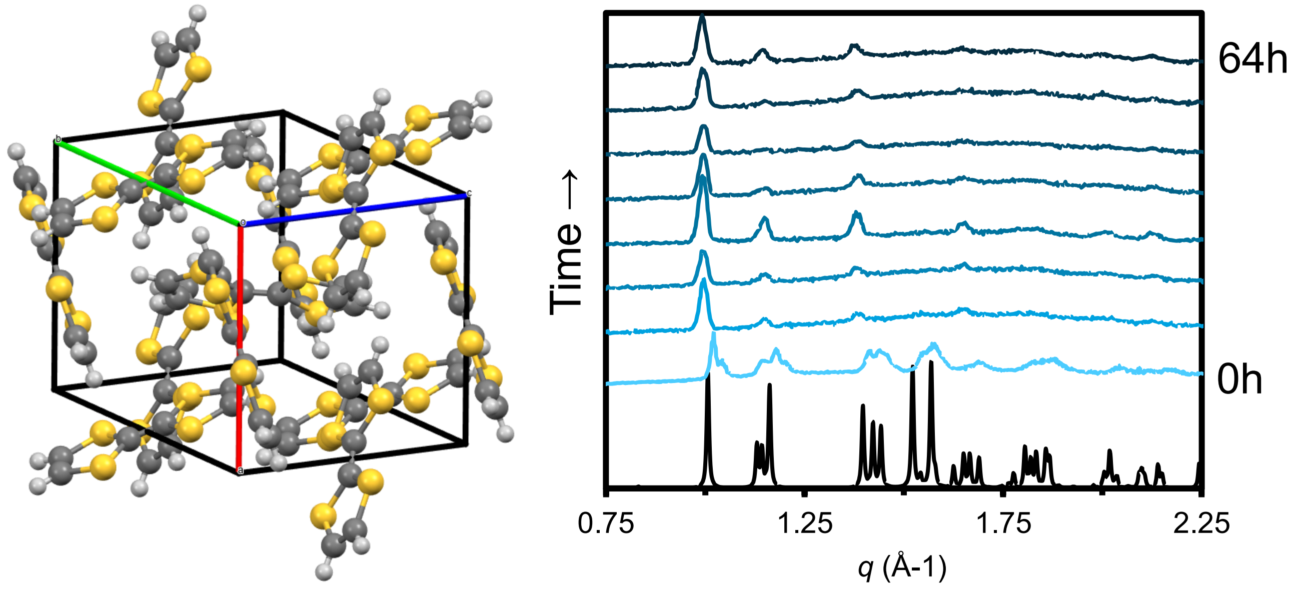

Figure S3: Crystal structure of β-TTF (CCDC code: BDTOLE02) and powder diffraction patterns of films annealed from 0 to 64 hours. The black spectrum is the simulated β-TTF diffraction pattern calculated in Mercury using the aforementioned structure.

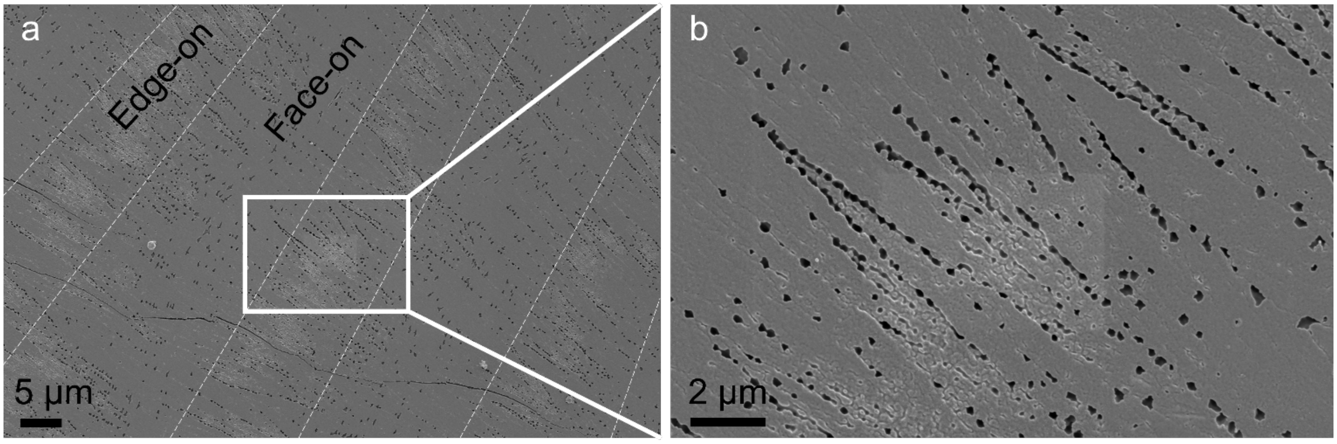

Figure S4: a) SEM image of banded TTF film melt processed and then held at 40 °C in a sealed vial for 4 hours. Dashed white lines roughly define the border between edge-on and face-on bands. b) expansion of an edge-on band from the SEM image in a).

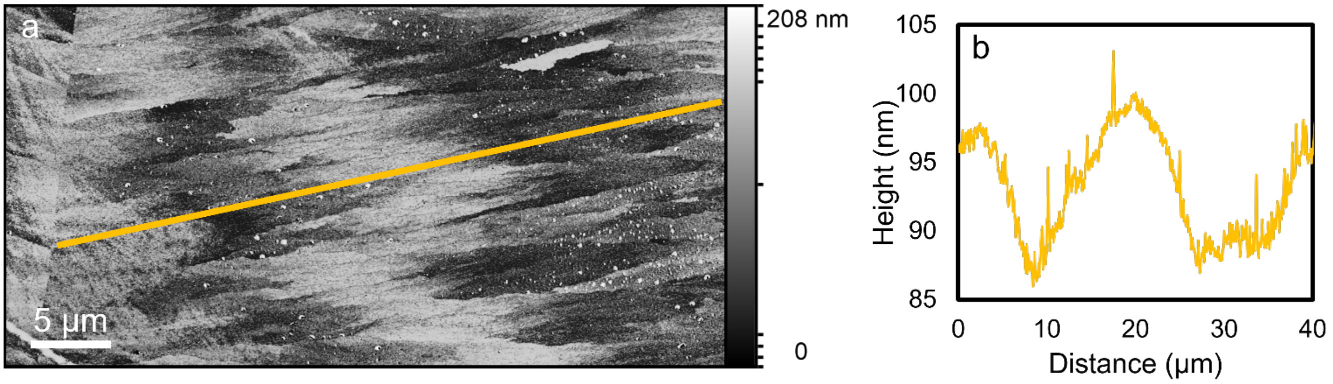

Figure S5: a) AFM heightmap of banded TTF spherulite covering an area of four bands. b) A height profile of the same color line present in a).

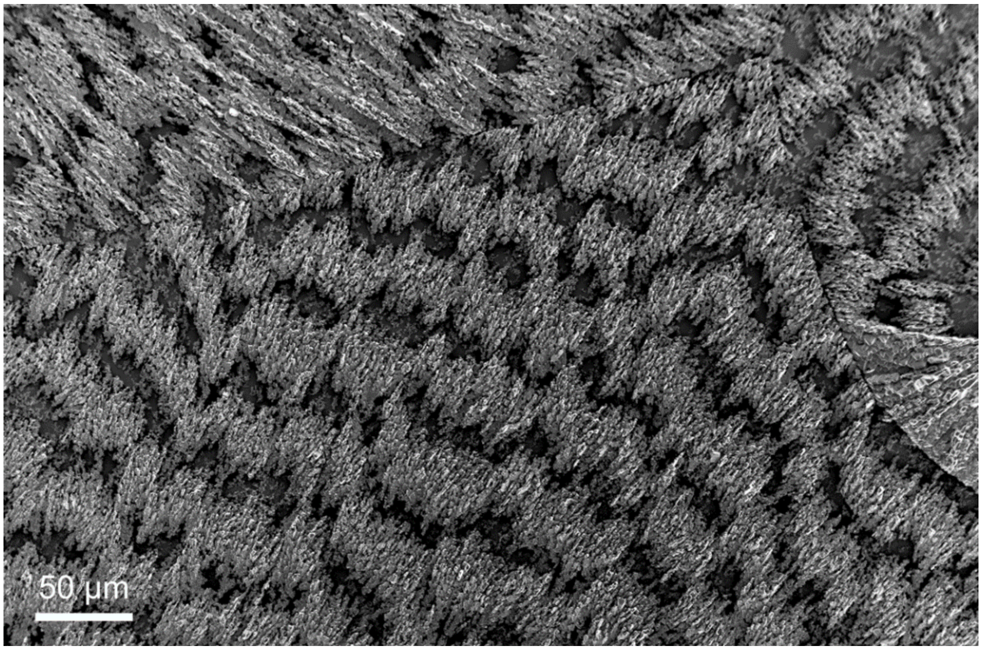

Figure S6: SEM of TTF sample methanol solvent vapor annealed for 64 h.

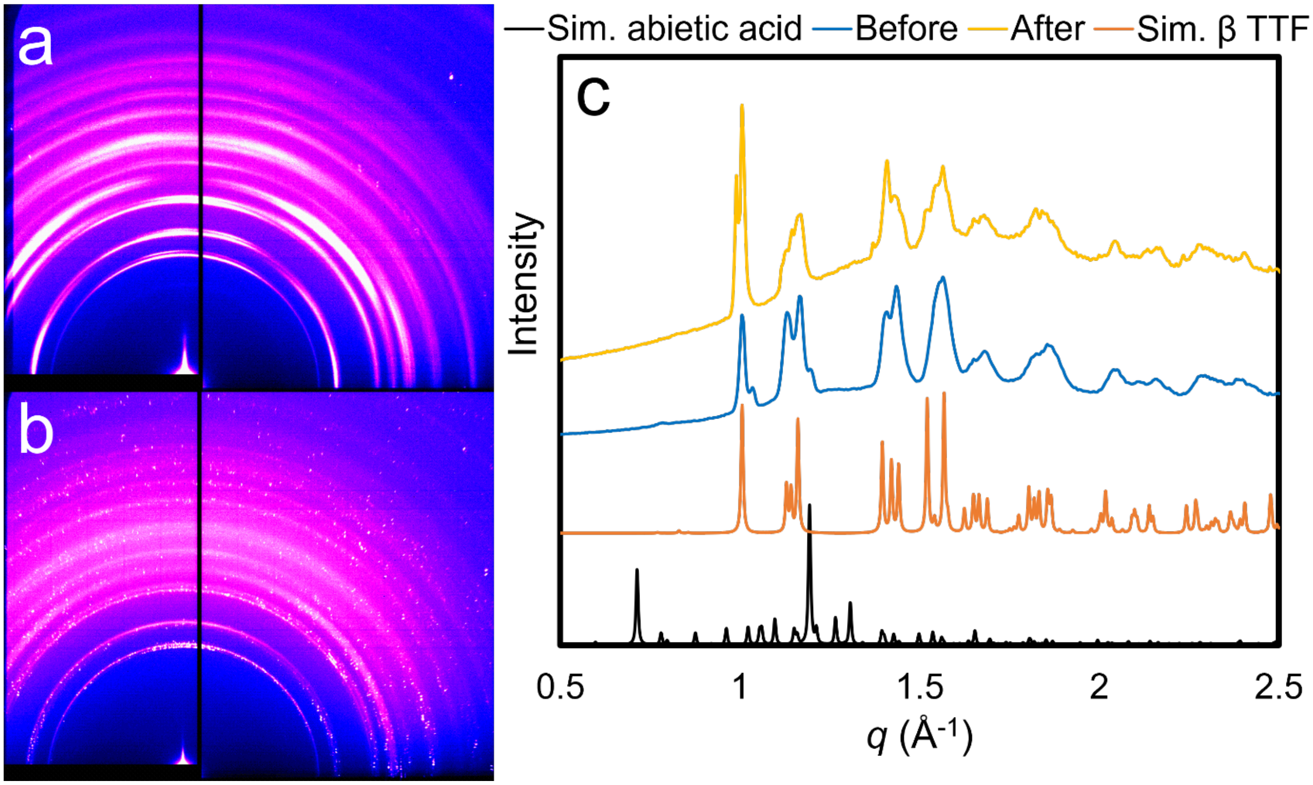

Figure S7: a) GIWAX diffraction pattern of as processed TTF banded spherulite film. b) GIWAXS diffraction pattern of TTf banded spherulite mehtnaol solvent vapor anneaed for 24 hours. c) Azimuthally integrated GIWAXS difractino patterns of a) and b) compared to Mercury simulated patterns of β-TTF and abietic acid (CCDC refcode: YAWMOY10).

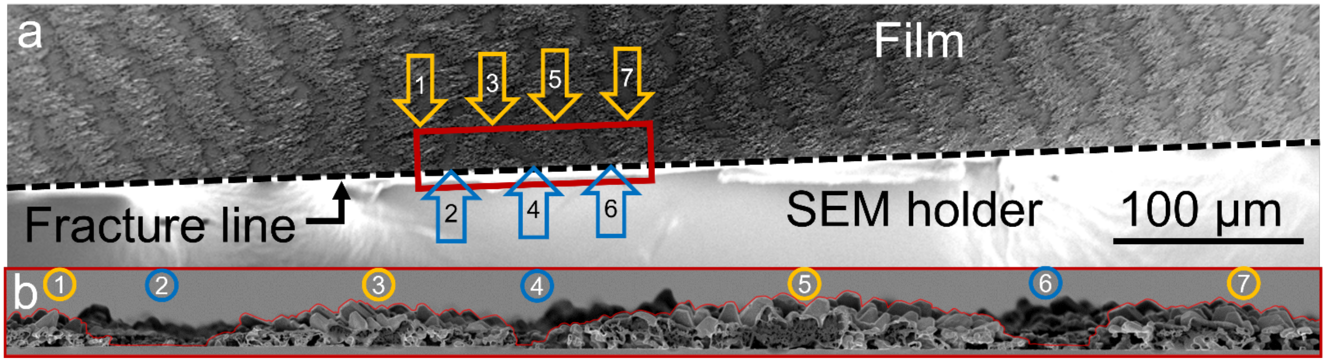

Figure S8: a) Top down SEM of area images in Fig 2f labelled with numbered arrows indicating ridges and valleys. The orange, odd-numbered arrows indicate the four ridges present in the cross-section, and the blue, even numbered arrows indicate valleys. b) The cross section from Fig 2f with crystallite layer outlined in red, and colored labels corresponding to the arrows in part a).

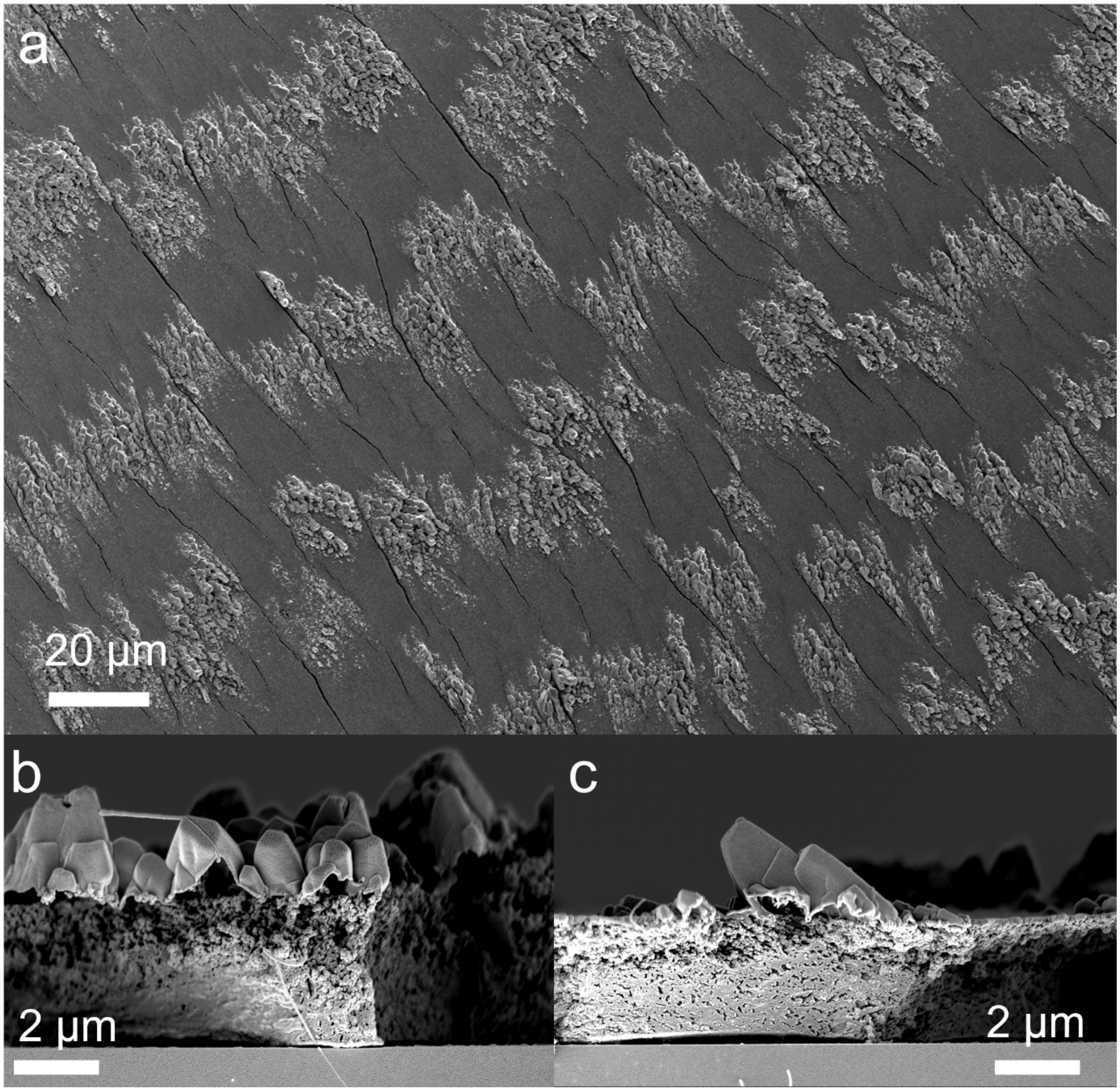

Figure S9: a) Top down SEM showing different crystallite tilts between bands of a TTF film where liquid methanol wet across the film surface for 4 seconds before rapidly air drying. b-c) Cross sectional SEM of the same film.

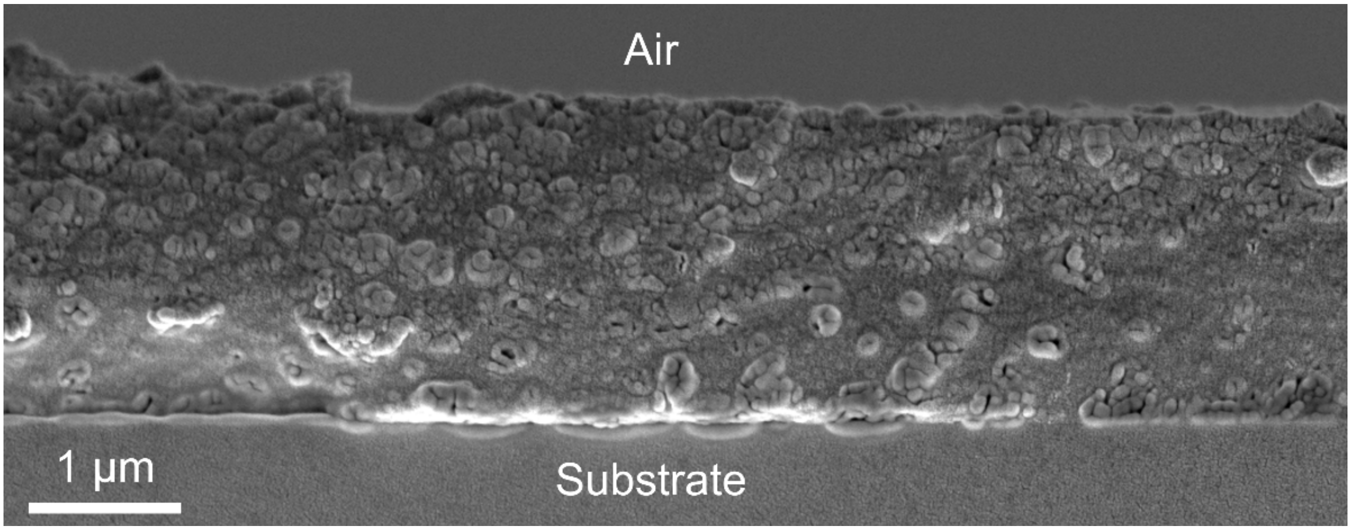

Figure S10: SEM cross-section of TTF banded spherulite film where spherulitic growth is parallel to the view, perpendicular to the cross-section cut.

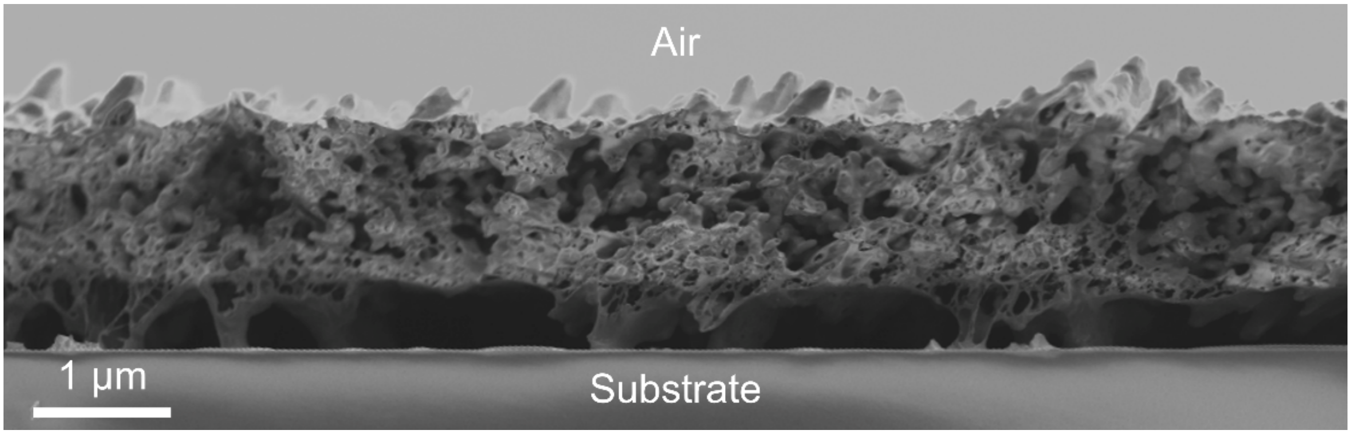

Figure S11: SEM cross-section of a TTF banded spherulite film solvent vapor annealed for 8 hours.

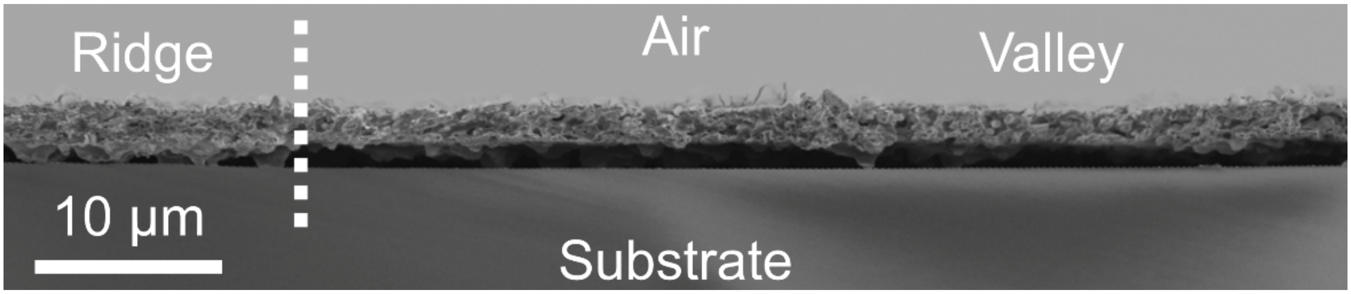

Figure S12: SEM cross-section of a TTF banded spherulite film solvent vapor annealed for 2 hours. This image shows the area between bands where recrystallization occurs. The film interior appears to be suspended above the substrate due to crystallite growth downward from the bottom surface. The dashed white line indicates the approximate edge between a ridge and a valley.

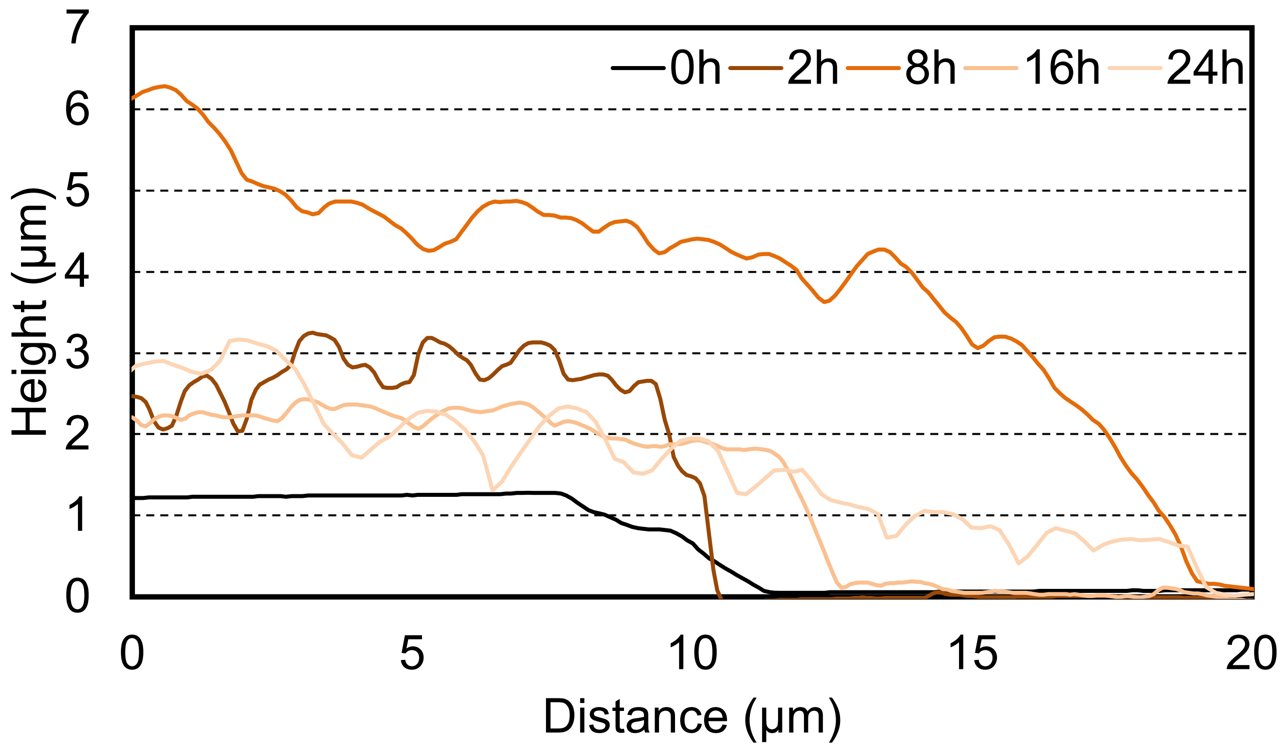

Figure S13: AFM height profiles extracted from samples solvent vapor annealed for 0, 2, 8, 16, and 24 hours. A razor was used to scrape the TTF film away prior to scanning so the glass substrate could be used as a reference height (Figure S14), except in the case of the 24-hour sample, where glass was exposed between bands (Figure S15).

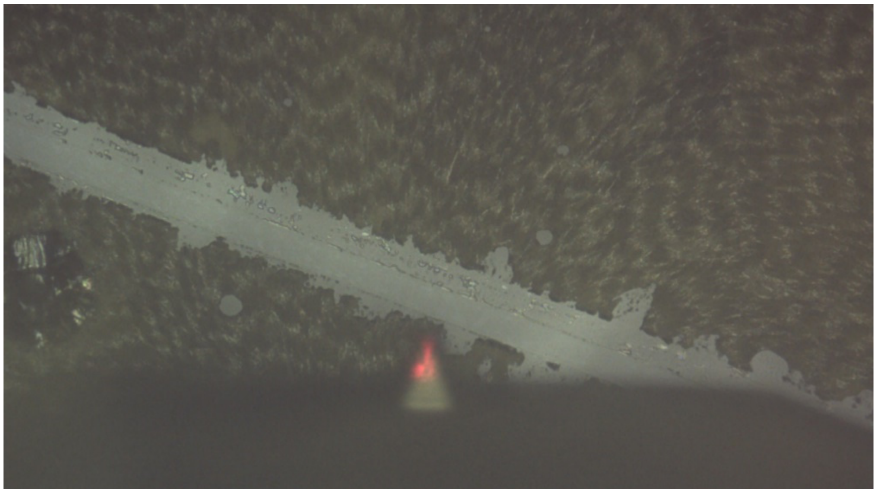

Figure S14: Optical micrograph taken in AFM software of a TTF film solvent vapor annealed for 16 hours. Portions of the film were removed in lines with the edge of a razor blade. The triangle with a red laser spot is the AFM cantilever.

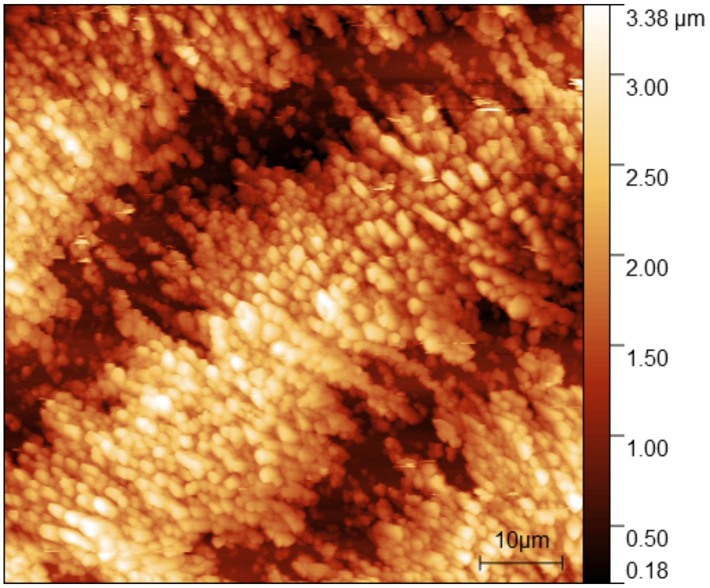

Figure S15: AFM height map of a TTF film solvent vapor annealed for 24 hours.

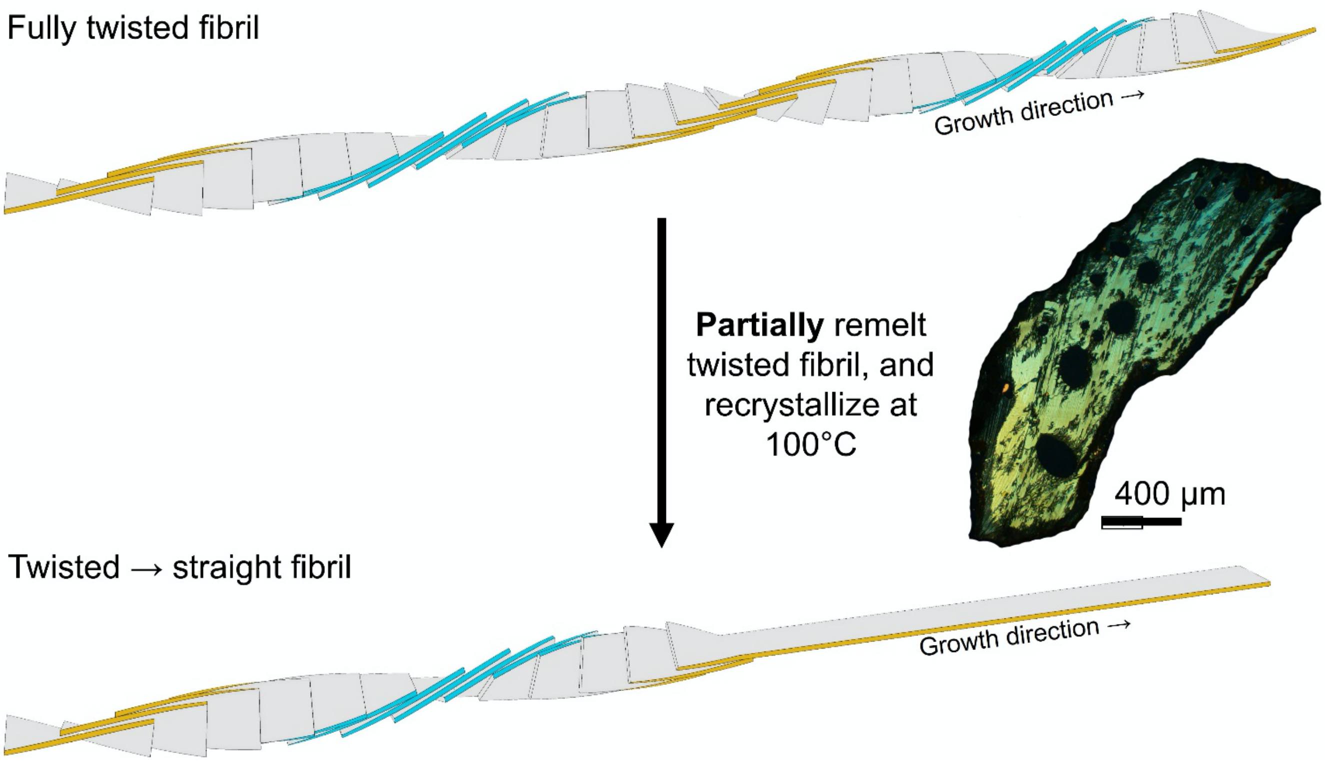

Figure S16: Scheme for growing "single crystal" from a twisted fibril. First, a banded spherulite film was made, then partially remelted by holding the sample near the melting point of TTF. Once the banded spherulite was partially remelted, the film was held at 100°C, causing a "single crystal" to nucleate from remaining banded spherulite edge. Once the single crystal formed, the banded portions of the film were scraped away, leaving a single crystal which was analyzed via grazing incidence x ray diffraction (POM inset).

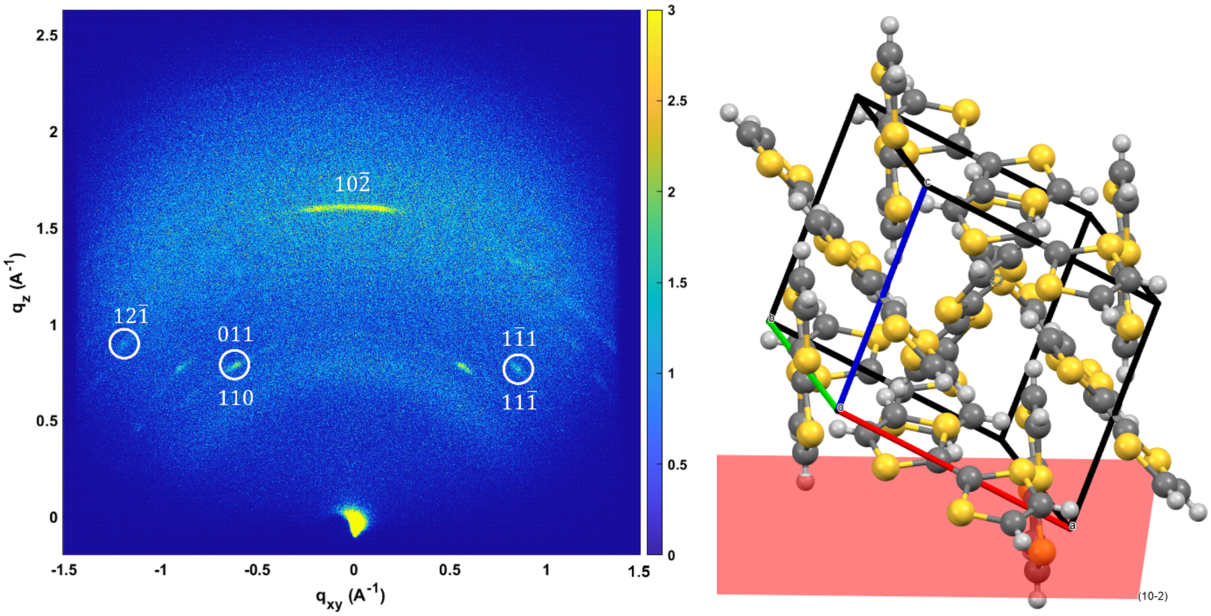

Figure S17: Indexed grazing incidence x ray diffraction pattern of β TTF “single crystal” described in Figure S16.

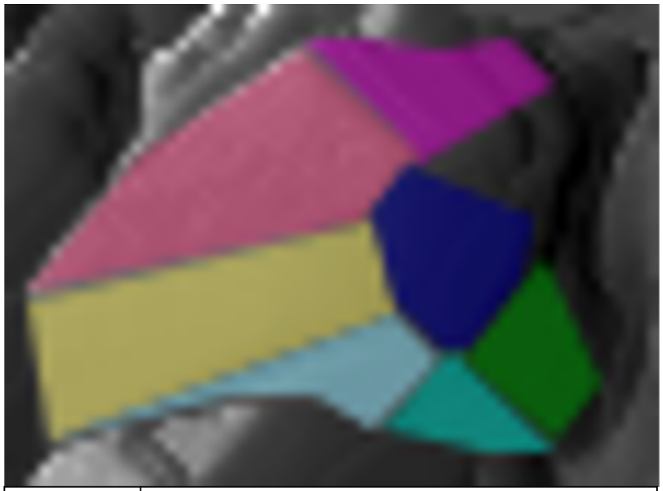

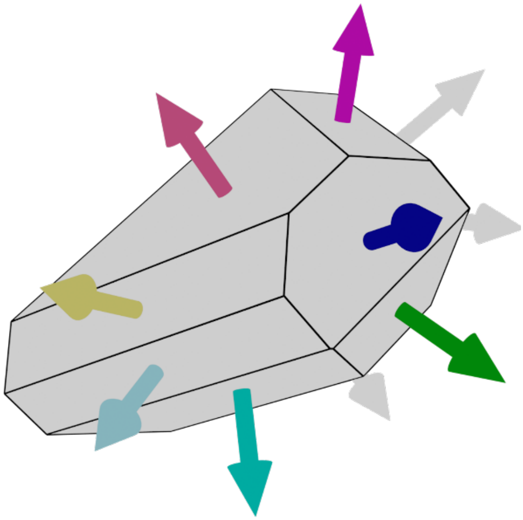

	Vector coordinates		
Facet	x	y	z
Dark Blue	0.53569872	-0.04407264	0.84325826
Green	0.76205916	-0.31439268	0.56605926
Teal	0.29415649	-0.80818356	0.51020711
Light Blue	-0.0955071	-0.63500027	0.76658532
Yellow	-0.49330529	0.0741284	0.86669191
Pink	-0.52531101	0.52165873	0.67225033
Purple	0.00989537	0.79435535	0.60737275

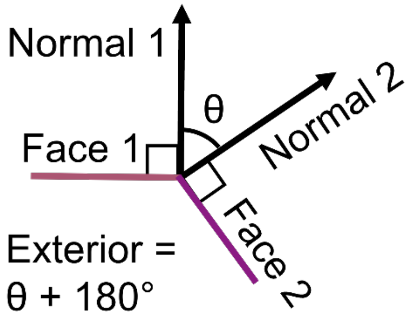

Actual	Dark Blue	Green	Teal	Light Blue	Yellow	Pink	Purple
Dark Blue	0	214.022	222.645	212.629	226.659	210.63	216.047
Green	214.02174	0	222.645	241.505	269.138	286.016	266.638
Teal	222.64527	222.645	0	212.629	258.453	288.205	293.288
Light Blue	212.62928	241.505	212.629	0	226.659	257.193	273.669
Yellow	226.65901	269.138	258.453	226.659	0	210.63	236.637
Pink	210.62968	286.016	288.205	257.193	210.63	0	216.047
Purple	216.04691	266.638	293.288	273.669	236.637	216.047	0
Calc	010	111	001	-111	-110	-11-1	01-1
010	0	234.972	259.634	244.362	245.322	259.847	245.057
111	234.97231	0	224.643	254.372	288.042	314.82	288.577
001	259.63423	224.643	0	227.302	271.6	314.38	324.691
-111	244.36207	254.372	227.302	0	224.298	267.078	291.768
-110	245.32176	288.042	271.6	224.298	0	222.78	254.273
-11-1	259.84725	314.82	314.38	267.078	222.78	0	221.559
01-1	245.05657	288.577	324.691	291.768	254.273	221.559	0

Figure S19: Angles between faces were measured using the facet measurement function of *Gwyddion* AFM analysis software. By quantifying the vector normal to each facet of a single crystallite, the exterior angles between all facets were derived from the vector dot product equation. Using *Microsoft Excel*, the angles between facets were tabulated and then quantitatively compared to all possible combinations of faces expected in the *Mercury* BFDH calculator (10 faces, 7 selected, 604,800 possible combinations). By calculating the percent difference between experimental AFM data and the theoretical exterior angle between planes from the single crystal structure, using the *Excel* solver, a difference minimized set of faces was found.
